# Role of the TGF-β cytokine and its gene polymorphisms in asthma etiopathogenesis

**DOI:** 10.3389/falgy.2025.1529071

**Published:** 2025-01-30

**Authors:** Jacek Plichta, Michał Panek

**Affiliations:** Department of Internal Medicine, Asthma and Allergology, Medical University of Lodz, Lodz, Poland

**Keywords:** TGF-β, asthma, single nucleotide polymorphism, genomics, gene polymorphism, pathogenesis

## Abstract

Transforming growth factor beta (TGF-β) is a pluripotent cytokine expressed by all cells of the human body which plays important roles in maintaining homeostasis and allowing for proper individual development. Disturbances in TGF-β signaling contribute to the development of many diseases and disorders, including cancer and organ fibrosis. One of the diseases with the best-characterized correlation between TGF-β action and etiopathogenesis is asthma. Asthma is the most common chronic inflammatory disease of the lower and upper respiratory tract, characterized by bronchial hyperresponsiveness to a number of environmental factors, leading to bronchospasm and reversible limitation of expiratory flow. TGF-β, in particular TGF-β1, is a key factor in the etiopathogenesis of asthma. TGF-β1 concentration in bronchoalveolar lavage fluid samples is elevated in atopic asthma, and TGF-β expression is increased in asthmatic bronchial samples. The expression of all TGF-β isoforms is affected by a number of single nucleotide polymorphisms found in the genes encoding these cytokines. Some of the SNPs that alter the level of TGF-β expression may be associated with the occurrence and severity of symptoms of asthma and other diseases. The TGF-β gene polymorphisms, which are the subject of this paper, are potential diagnostic factors. If properly used, these polymorphisms can facilitate the early and precise diagnosis of asthma, allowing for the introduction of appropriate therapy and reduction of asthma exacerbation frequency.

## Introduction—TGF-β

Transforming growth factor β (TGF-β) is a pluripotent cytokine expressed by all somatic human cell types (1), which plays several important roles in maintaining homeostasis (2). TGF-β occurs in three isoforms (TGF-β1, 2 and 3). All forms of TGF-β are receptor ligands of three receptor types (TβRI, II and III), have similar biological activity and participate in processes such as regulation of proliferation, migration, differentiation and apoptosis (3).

TGF-β regulates cell proliferation, contributes to epithelial-to-mesenchymal transition (EMT), regulates immune cell function by modulating immune responses, contributes to the conversion of fibroblasts to myofibroblasts, and causes overproduction of the extracellular matrix (ECM) in tissues undergoing fibrosis (4). TGF-β stimulates expression of proteins such as collagens, basal lamina proteins, and ECM proteins (5). Additionally, in the early stages of tissue fibrosis, TGF-β stimulates myofibroblasts and other stromal cells to increase the synthesis of collagen-crosslinking enzymes (6). Furthermore, TGF-β decreases the synthesis of matrix-depleting proteins, such as matrix metalloproteinases. Ultimately, the increased synthesis of matrix proteins and decreased activity of matrix metalloproteinases due to TGF-β activity contribute to the remodelling of epithelial tissue and may result in fibrosis (7–9).

**Figure 1:**
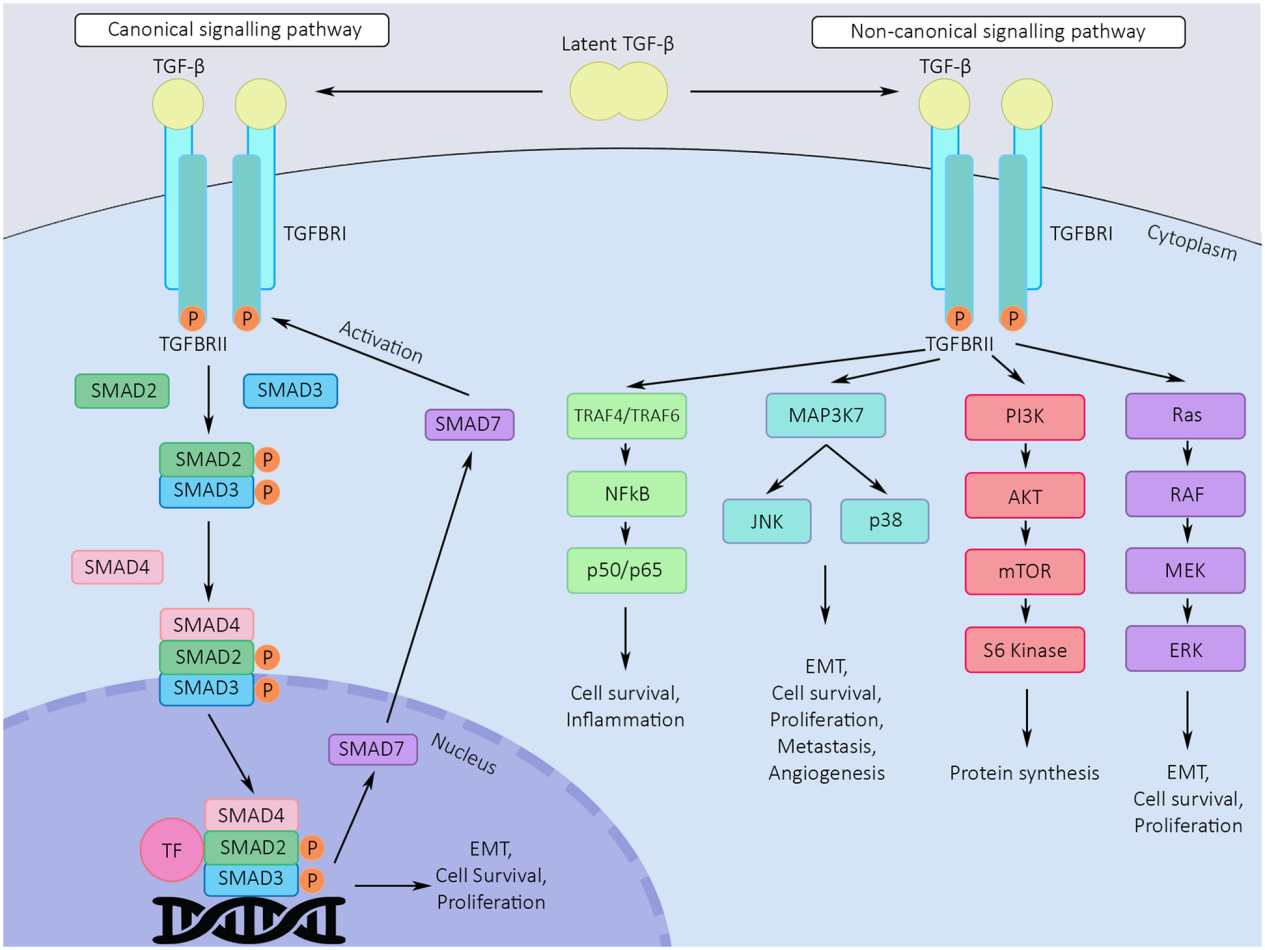
Canonical and non-canonical TGF-β signalling pathways. TGF-β is secreted in a latent, inactive form and activated in an integrin-dependent manner. Active TGF-β binds to TGF-β type II receptors (TGFBRII), which are transmembrane receptors with serine/threonine kinase activity. TGF-β-TβRII interactions lead to the recruitment and phosphorylation of TGF-β type I receptors (TGFBRI). In the canonical signalling pathway, upon activation, TβRIs phosphorylate SMAD2 and SMAD3, which then assemble into heterodimers and trimers with SMAD4. Complete SMAD protein complexes translocate to the nucleus, interact with transcription factors and influence the expression of TGF-β target genes. SMAD7 inhibits TGFBRII, acting as a regulatory factor in the signalling pathway. In the non-canonical signalling pathway, phosphorylated TGFBRII instead activates one of several alternative pathways, including the ERK, JNK, p38 and PI3K-Akt pathways. P, phosphorylation; TF, transcription factor; EMT, epithelial to mesenchymal transition.

The canonical TGF-β signalling pathway is activated by the binding of TGF-β ligands to their receptors. TGF-β is secreted in a latent, inactive form (L-TGF-β) and activated in an integrin-dependent manner (10). Active TGF-β binds to TGF-β type II receptors (TβRII), which are transmembrane receptors with serine/threonine kinase activity. TGF-β-TβRII interactions lead to the recruitment and phosphorylation of TGF-β type I receptors (TβRI) (11). Upon activation, TβRIs phosphorylate SMAD2 and SMAD3, which then assemble into heterodimers and trimers with SMAD4. Complete SMAD protein complexes translocate to the nucleus and influence the expression of TGF-β target genes. SMAD3 and SMAD4 bind DNA directly, with low affinity, and require the presence of other transcription factors to fully regulate target gene expression. SMAD2 does not bind DNA directly (12). Target genes regulated by TGF-β include plasminogen activator inhibitor-1 (*PAI-1*) and *Smad7*, which acts as an inhibitor of TGF-β/SMAD signalling by labelling activated TβRI for proteasomal degradation (13). Figure 1 is a simplified visual representation of the TGF-β signalling scheme.

Dysfunction of TGF-β signalling influences the development of many diseases and disorders, including cancer and organ fibrosis (14, 15). Excessive TGF-β activity promotes organ fibrosis by increasing connective tissue growth factor (CTGF) expression, inhibiting extracellular matrix (ECM) degradation, and increasing collagen synthesis. TGF-β also induces fibroblast proliferation and differentiation (16). The induction of organ fibrosis by TGF-β is mediated by Smad-dependent pathways, which directly increase the transcription of genes involved in ECM production, and Smad-independent pathways, including MAP kinase pathways (17).

## Introduction—asthma

Asthma is the most common chronic inflammatory disease of the respiratory tract (18), characterized by bronchial hyperresponsiveness to a number of factors, which leads to bronchoconstriction and reversible airflow limitation. Asthma is a heterogeneous disease with a significant degree of variability in phenotypes (clinical symptoms in conjunction with patient characteristics, such as age of asthma onset, clinical response to pharmacological therapies and presence of comorbidities) and endotypes (pathophysiological mechanisms leading to the development of the disease) (19). Characteristic symptoms of asthma include wheezing, shortness of breath, cough, and chest tightness. Symptoms vary in severity and frequency and occur mainly during disease exacerbations (20). Bronchial asthma can be divided into two main endotypes based on the presence of inflammatory responses mediated by T helper type 2 (Th2) lymphocytes. The two classically recognized endotypes are type 2 (eosinophilic) asthma and non-type 2 (non-eosinophilic) asthma (21). The discovery that innate lymphoid cells type 2 (ILC2) are capable of releasing Th2 cytokines has led to a more precise classification of asthma endotypes into T2-high, T2-low, and non-T2 (22).

The clinical picture of asthma depends on complex gene-gene and gene-environment interactions. These components make asthma a dynamic disease, and its classifications can change both due to treatment and independently of it. At the outset of asthma development, airway hyperresponsiveness to normally harmless antigens, such as pollen or mites, causes epithelial cells to release inflammatory mediators, including many interleukins (23). Inflammatory mediators trigger an immune activation cascade that leads to activation and recruitment of ILC2s, mast cells, Th2 cells, eosinophils, and dendritic cells to the site of inflammation (24). Repeated exposure to allergens can lead to chronic airway inflammation and airway remodelling, which ultimately leads to partial loss of respiratory function (25). Airway remodelling in asthma includes subepithelial fibrosis, abnormal extracellular matrix deposition, goblet cell proliferation, smooth muscle and mucous gland hypertrophy, and epithelial damage (26).

The disease develops under the influence of variables that, according to the Global Strategy for Asthma Management and Prevention (GINA) report (27), can be divided into exacerbation-inducing (inducing disease exacerbations in patients with pre-existing asthma) and asthma-causing (causing the development of asthma in patients without pre-existing asthma). According to the current GINA guidelines, the Asthma Control Questionnaire (ACQ) (28) or the Asthma Control Test (ACT) (28) can be used to assess symptoms. The severity of the disease is assessed not based on the severity of symptoms before treatment, but only after several months of treatment, when the level of severity and the intensity of treatment required to achieve and maintain asthma control are established (29).

Asthma has also been studied in the context of pharmacogenomics. In a systematic review of pharmacogenomic studies, García-Menaya et al. focused on four genes with the potential to be implemented in pharmacogenomics for asthma therapy. *FCER2* was shown to be related to inhaled corticosteroid (ICS) response, *ABCC1* and *LTC4S* were related to anti-leukotriene agents, and *ADRB2* was related to beta-agonist response (30). In another systematic review which examined 30 studies involving over 6,000 subjects, variant rs1042713 of the *ADRB2* gene was consistently associated with response to long-acting beta-agonists (LABA) (31). Another systematic review focused on the main findings of pharmacogenetic studies of pediatric asthma published in 2018–2019. The findings of the studies discussed in this review led to the validation of previously established gene-treatment response associations in asthma, and more importantly—allowed the identification of novel associations (*PRKG1, DNAH5, IL1RL1, CRISPLD2, MMP9, APOBEC3B-APOBEC3C, EDDM3B,* and *BBS9*). Critically, many of the results were not consistent between studies, underlining the need for large studies in diverse populations and a harmonization of treatment response definitions (32). It is important to note, that despite over 35 guidelines being published for pharmacogenomics-based optimization of therapy by the Clinical Pharmacogenetics Implementation Consortium (CPIC), there are no guidelines for asthma (33). In spite of much effort put towards researching pharmacogenomic biomarkers for asthma therapy, more compelling, robust clinical evidence is required for diagnostic implementation.

### TGF-β and its signaling pathways in asthma etiopathogenesis

TGF-β, in particular TGF-β1, is a key factor in the etiopathogenesis of asthma. TGF-β1 levels in bronchoalveolar lavage fluid (BALF) are elevated in atopic asthma (34, 35), and TGF-β expression is increased in bronchial tissue (36), sputum (37) and serum (38) samples from asthmatics. TGF-β2 levels have been found to be significantly increased in airway tissues extracted from patients with severe asthma (39). Peripheral blood neutrophils from asthmatic patients were found to express higher levels of TGF-β than those of non-asthmatic subjects (40). Human bronchial fibroblasts (HBF) from asthmatic patients also exhibit increased secretion of TGF-β1, both when unstimulated (41, 42) and when stimulated with interleukin-13 (IL-13), a cytokine and central mediator of asthmatic inflammation (43). Lastly, a relationship between airway TGF-β levels and asthma severity has been suggested by several researchers (44–46).

Bronchial epithelial cells and eosinophils are the main source of TGF-β in the airways of asthmatics (47). TGF-β stimulates airway smooth muscle proliferation and extracellular matrix (ECM) deposition by activated fibroblasts, ultimately leading to structural changes in the airways. Furthermore, TGF-β1 promotes airway smooth muscle shortening and hyperresponsiveness by enhancing excitation-contraction coupling (48). Multiple studies using animal models of asthma have pointed towards the role of TGF-β in the remodelling of asthmatic airways. McMillan et al. have shown that anti-TGF-β antibody treatment prevents the progression of airway remodeling using a murine model of prolonged allergen challenge. Furthermore, anti-TGF-β antibody treatment was shown to limit activation and expression of Smad 2, while upregulating the expression of inhibitory Smad7 (49). Another study used a murine model of ovalbumin sensitization and challenge to assess the effects of isoform-specific anti–TGF-β antibodies on asthma-associated airway remodelling. Antibodies against TGF-β1 and TGF-β2 were shown to inhibit ovalbumin-induced subepithelial collagen deposition, while anti-TGF-β1 antibodies additionally limited TGF-β1-mediated fibroblast decorin (a protein which influences collagen fiber development) deposition (50).

**Figure 2:**
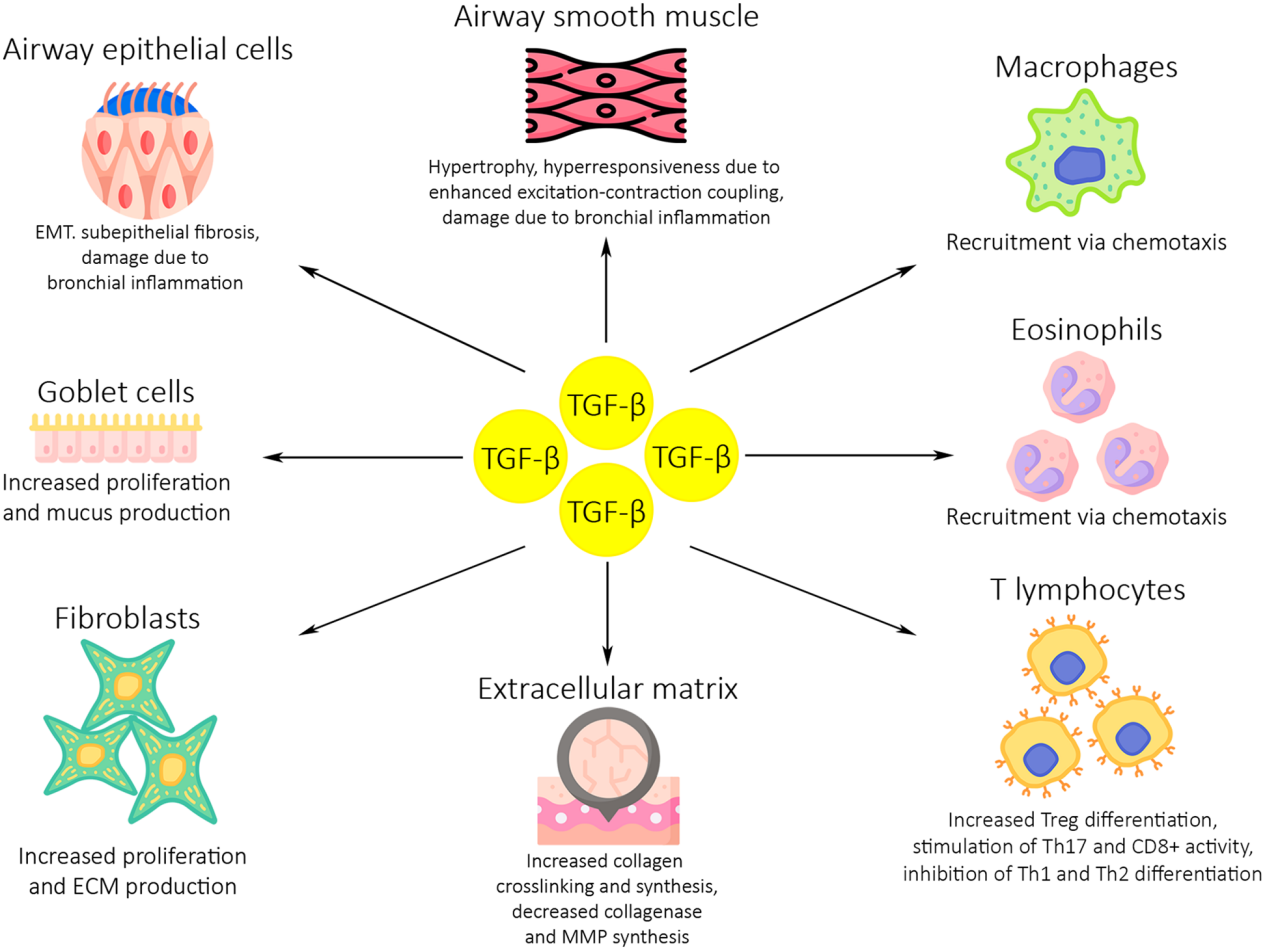
The role of TGF-β in asthma development and exacerbations. Dysregulated TGF-β activity induces inflammation and airway remodelling in the asthmatic airways through several mechanisms. High levels of TGF-β expression lead to an increase in macrophage, eosinophil and T lymphocyte activity and proliferation. An increase in proliferation and activity is also seen in fibroblasts and goblet cells, which leads to higher levels of mucus production and ECM deposition. Airway epithelium and smooth muscle are damaged due to bronchial inflammation, resulting in subepithelial fibrosis and bronchial hyperresponsiveness. ECM, extracellular matrix; MMP, matrix metalloproteinases; EMT, epithelial to mesenchymal transition.

Several studies carried out on human bronchial tissues have also suggested that TGF-β signalling has a significant role in asthmatic airway remodelling. In one such study, acute allergen challenge led to an increase in expression of TGF-β2, Smad 2/3 and Smad 4 in airway epithelial cells and leukocytes. Furthermore, the same study indicated that increased TGF-β2 expression may be responsible for an increase in the deposition of tenascin, an ECM glycoprotein, in bronchial tissues (51). Another, similar study has shown a correlation between higher connective tissue growth factor (CTGF) levels and increased basement membrane thickness. CTGF expression is induced by TGF-β1 (52). Human bronchial epithelial cells exposed to TGF-β1 have exhibited an upregulated expression of disulfide-isomerase, a profibrotic enzyme, compared to non-stimulated cells (53). Wnuk et al. examined the effects of TGF-β on the balance between the profibrotic Smad2/3 and antifibrotic Smad1/5/9 pathways and myofibroblast formation in human bronchial fibroblast populations from asthmatic and non-asthmatic patients. They showed that TGF-β strongly enhanced the Smad2/3 pathway activity, while diminishing activity of the Smad1/5/9 pathway in fibroblasts from asthmatic patients. Additionally, these changes in signalling pathway activity were correlated with enhanced fibroblast-to-myofibroblast transition (FMT). Interestingly, activation of the Smad1/5/9 through administration of BMP7 was shown to prevent FMT and downregulate profibrotic gene activity in fibroblasts from asthmatic patients (8). Figure 2 depicts the effects of TGF-β on asthma development and exacerbations.

The role of TGF-β in asthma is not limited to airway remodelling. In CD4+ CD25− T cells, TGF-β induces expression of the *Foxp3* gene upon stimulation of T cell receptors. Expression of the *Foxp3* gene leads to the differentiation of regulatory lymphocytes (Treg) with immunosuppressive activity (54). Importantly, TGF-β is also produced by Treg lymphocytes. TGF-β in combination with IL-6 also stimulates the proliferation of Th17 lymphocytes, which contribute to the recruitment of neutrophils to the airways, which ultimately leads to the development of an eosinophilic Th2-type inflammatory reaction (55, 56). TGF-β inhibits the differentiation of Th1 and Th2 lymphocytes by silencing the transcription factors T-beta and GATA3 (57). This indicates a dual role of TGF-β in the Th2-type inflammatory reaction. In summary, TGF-β stimulates the activity of NK, Treg, Th17, and CD8+ T cells while inhibiting the differentiation of Th1 and Th2 cells. TGF-β influences the immune system in the asthmatic airways by playing multifunctional roles in T cell differentiation and homeostasis (58).

SMAD proteins responsible for TGF-β signal transduction also influence the course of chronic airway inflammation. Mice with inactivated *Smad3* gene developed significantly less peribronchial fibrosis, smooth muscle proliferation and mucus production in the airways after allergen challenge compared to wild-type mice (59). Overexpression of SMAD2 in the epithelium may, on the other hand, increase subepithelial collagen deposition and smooth muscle hyperplasia without causing airway inflammation (60). Studies conducted on asthmatics treated with mepolizumab, a monoclonal antibody against IL-5, clearly indicate a link between the level of eosinophil proliferation in the airways and the expression of TGF-β and asthmatic airway remodelling. Patients who received mepolizumab for 3 months showed significantly lower levels of eosinophils in bronchoalveolar lavage (BAL). Importantly, the reduction in BAL eosinophils led to a significant reduction in BAL TGF-β1 levels in this group of patients. The reduction in eosinophil and TGF-β1 levels was accompanied by a reduction in ECM protein levels in the airways (61). This suggests that anti-IL5 therapy had an inhibitory effect on airway remodelling.

Allergic airway inflammation and airway hyperresponsiveness (AHR) are hallmarks of asthma crucial for the pathogenesis of this disease. With respect to the immunology of asthma pathogenesis, Th2 cytokines such as IL-4, IL-5, IL-9, and IL-13 are considered key mediators in the development of airway inflammation and AHR (62, 63). IL-13 is currently considered to be a central mediator in the development of allergic asthma (64). TGF-β1 is a key mediator in the development of IL-13-mediated asthma phenotypes. TGF-β1 is thought to be involved in the phenotypic changes of myofibroblasts, resulting in airway remodelling due to overexpression of IL-13 in TG(+) mice (65). These findings suggest that TGF-β1 signalling pathways are involved in the development of AHR.

In summary, in asthmatic airways, TGF-β1 causes immunosuppression of Th1 and Th2 lymphocytes, stimulates the proliferation of Th17 lymphocytes, is a chemotactic factor for macrophages, fibroblasts and eosinophils (after the introduction of allergens to the airways), stimulates the synthesis of ECM proteins while inhibiting the expression of collagenase and matrix metalloproteinases, and stimulates the proliferation of bronchial myocytes (66).

## Review methodology

For the following section (“TGF-β gene polymorphisms and asthma etiopathogenesis”), literature research has been performed in the PubMed and Google Scholar databases using the following keywords: “snp” or “polymorphism” and “tgf” or “tgfb” or “tgf beta” and “asthma”. The inclusion criteria were as follows: (1) studies focused on the effects of transforming growth factor-β gene polymorphisms on asthma risk and severity, and (2) articles written in English. The exclusion criteria were as follows: (1) reviews, editorials, opinion articles or case studies; (2) animal or *in vitro* model studies; (3) papers that mentioned but did not directly study TGFB gene polymorphisms. A stepwise evaluation of each position in the search results according to the inclusion and exclusion criteria has been conducted based on the title, the abstract, and lastly the full text. Each paper has been independently evaluated by both authors.

### TGF-β gene polymorphisms and asthma etiopathogenesis

The *TGFB1* (Transforming Growth Factor Beta 1) gene is located on 19q13.2 and encodes a secreted protein (44 kDa) of 390 amino acids. The protein, also named Transforming growth factor beta 1, is secreted in a latent form and must be activated in an integrin-dependent manner (67). The *TGFBR3* (Transforming growth factor beta receptor 3) gene is located on 1p33-p32 and encodes a protein (300 kDa) of 849 amino acids, of the same name, with a single transmembrane domain and a short section of the intracellular domain (68).

The expression and activity of all TGF-β isoforms is influenced by a number of single nucleotide polymorphisms (SNPs) occurring in the genes encoding these cytokines. SNPs are substitutions of single nucleotides at a specific genomic position. SNPs do not necessarily change the encoded protein due to the genetic code being degenerated. If a SNP is defined by a C being replaced with a T in some individuals, those two variations (C/T) are defined as alleles of that SNP. Some SNPs change the level of TGF-β expression and may be associated with the occurrence and intensity of asthma and other diseases (69, 70). Polymorphic forms of the *TGF*β genes may significantly contribute to the development of asthma, induce disease progression and its complications, and have a clinically significant impact on symptom control (71).

Currently, the exact role of SNPs in the pathogenesis of asthma is the subject of widespread discussion, and numerous studies are being conducted in this area. It should be noted that the studies are being conducted on populations of disparate characteristics and sizes, thus not all results are reproduced. It should be noted that many studies have demonstrated and confirmed the functional role of *TGF*β SNPs in asthma (72–74). From the point of view of both basic science and clinical applications, gaining an understanding of the influence of these SNPs on TGF-β signalling pathways in asthma is highly important.

#### −509C/T (rs1800469)

A growing body of evidence suggests that alterations in TGF-β signalling pathways may lead to the development of allergic disease, in which IgE specific for normally harmless substances is produced. IgE binds to the surface of mast cells, and cross-linking of IgE through allergen binding results in mast cell degranulation and allergic symptoms (75). One of the early findings suggesting a role for TGF-β in atopy came from a study of 20 families with an asthmatic proband and 10 families without asthma. This study identified a polymorphism in the *TGFB1* promoter (a sequence bound by proteins to initiate RNA transcription) 509C>T (rs1800469), meaning the cytosine at position 509 in the *TGFB1* promoter sequence has been replaced with a thymine. The polymorphism was present in a homozygous form in 16 individuals and significantly associated with increased total IgE in a population of 47 unrelated individuals (76). Subsequent studies with larger study populations have shown that 509C>T was strongly associated with increased plasma TGF-β1 levels and was associated with the development of asthma (77, 78). An association of the 509C>T polymorphism with increased serum IgE levels or increased risk of asthma development was confirmed in some (78–84) but not all studies (85–88). These disparate results may stem from differences in the ethnicities, ages and sexes of populations recruited for these studies. For example, Hobbs et al. (76) recruited families of undetermined ethnicity from the National Jewish Medical and Research Center in the USA. Grainger et al. (77) recruited a population of post-menopausal (mean age 57 years) white British twins. Nagpal et al. (78) examined two cohorts of young Indian adults (mean ages 28 and 33 years for the two cohorts). Chiang et al. recruited 217 adult Taiwanese asthmatic subjects (79). Yucesoy et al. studied adult, Caucasian French Canadians exposed to diisocyanates (chemicals used in industrial processes, known to induce occupational asthma) (80). Wiśniewski et al. examined 247 adult Polish asthmatic patients (87). Lastly, Silverman et al. (86) examined DNA samples from a population of white North American adults. As asthma is a heterogeneous disease associated with a range of genetic and environmental factors, conducting studies on populations which differ ethnically and clinically (age, years from disease onset, disease severity) may lead to significant discrepancies in results. The T allele of rs1800469 in the *TGFB1* promoter region has also been found to be associated with elevated plasma TGF-β1 levels, elevated total IgE levels, and an increased risk of bronchial remodelling (89).

Whereas Salam et al. showed that within a group of 927 Hispanic and 2,096 non-Hispanic white children, those with the previously discussed −509TT rs1800469 genotype had a 1.8-fold increased risk of persistent asthma in California (90), de Faria et al. (91) suggested that there was no association between the presence of the −509C/T polymorphism and an increased risk of developing severe asthma in Brazilian children. A meta-analysis including a total of 12 case-control studies conducted in Chinese populations (92) suggested that the −509C/T polymorphism is associated with an increased risk of asthma. Subgroup analysis of these populations by age showed that the −509C/T polymorphism was strongly associated with the risk of asthma in Chinese children, but this polymorphism was not associated with asthma in adults. Subgroup analysis by asthma severity showed that the −509C/T polymorphism was associated with both mild to moderate asthma and severe asthma (92).

The −509C/T polymorphism locus is located in the promoter region of the *TGFB1* gene and may alter the activity of the *TGFB1* promoter-reporter and affect the interaction of the promoter region with transcription factor YY1, which ultimately leads to a change in the plasma TGF-β1 concentration (86). As the polymorphism is located in the promoter region, it does not affect the structure of the protein, but changes the amount of protein produced. The T allele is known to prevent AP1 from binding the promoter, increasing the levels of protein expression (93). Several studies have shown that the *TGFB1* −509C/T polymorphism has a statistically significant association with increased serum TGF-β1 levels in patients with asthma (76). Moreover, the −509T variant was found to be the *TGFB1* polymorphism with the strongest association to the severity of asthma (94). These results indicate that the presence of the −509C/T polymorphism may enhance the transcription of the *TGFB1* gene and may be a useful factor in the diagnosis of asthma.

In a meta-analysis conducted in 2016, 20 studies involving over 3,500 asthmatic patients were examined to analyse the association between TGF-β SNPs and asthma development risk. For the C509T polymorphism, a significant association was found both in overall analysis, and in subgroup analysis by ethnicity, for the Asian (*P* = 0.004, OR = 1.43) and Caucasian (*P* = 0.05, OR = 1.16) population. For the T869C polymorphism, a small but significant association was found in overall analysis (OR = 1.14, *P* = 0.03), but not in subgroup analysis by ethnicity. The authors point out limited sample size after subgrouping as an explanation for this discrepancy, once more emphasising the need for more studies on asthmatic populations from varied ethnic backgrounds (95).

#### +869T/C (rs1982073)

Sharma et al. conducted the first study of the association between *TGFB1* variants and disease exacerbation in people with asthma. This study examined two populations of children and showed that the T allele of the rs1982073 (+869T/C) polymorphism in *TGFB1* was associated with a lower risk of exacerbations (96, 97).

The +869T/C *TGFB1* polymorphism locus is in a domain which likely promotes the transport of newly synthesized protein to the endoplasmic reticulum (98). This variant may affect the function of TGF-β1, likely by affecting intracellular transport or the efficiency of cellular protein export. The SNP is located in the first exon of the gene, which allows it to impact the structure and function of the protein product (99). The +869T/C polymorphism may play an important regulatory role in *TGFB1* mRNA expression (100). The T allele has been shown to be associated with reduced *TGFB1* expression, while the C allele has been shown to exhibit 2.8-fold higher TGF-β1 secretion than the T allele (101). The 869T allele may result in lower efficiency of cellular protein export and lead to reduced protein production. The C allele has also been associated with elevated plasma TGF-β1 concentrations in Japanese patients with myocardial infractions (102). Studies have shown that the +869T/C polymorphism may also be involved in the modulation of asthma severity (91), indicating that this variant may influence asthma susceptibility and development and serve as a diagnostic factor in asthma.

#### Other polymorphisms

In 2022, a study was conducted on the association between SNPs in the TGFβ genes and the occurrence and asthma severity in a Polish population. It was found that there are four SNPs located in introns of the TGFβ2 gene (rs2796821, rs10779329, rs4903359 and rs11083616), the occurrence of which has a statistically significant association with the risk of asthma (66). In this study, data from 237 families with atopic asthma and 268 families without asthma were analysed. Statistical analysis confirmed previously published associations between many SNPs and asthma. In this study, very strong statistically significant differences in the occurrence of asthma were observed for rs2796821. The heterozygous (C/T) form of rs2796821increased the risk of asthma by 93%, while the homozygous C/C form increased the risk by 102%. Importantly, this analysis also allowed the discovery of new relationships between the occurrence of the C/C genotype for rs10779329 and the A/G genotype for rs4903359 in the *TGFB2* gene and a statistically significantly increased risk of asthma (66). The occurrence of the rs11083616 polymorphism was also found to be associated with bronchial obstruction, as well as increased airway wall thickness, which increases the risk of obstructive diseases (66).

Another study conducted on a Polish population showed that the T/C genotype of the rs8109627 polymorphism is significantly associated with an increased score in the ACT test, which is associated with better disease control—controlled asthma (7). Moreover, these studies indicated an association between the G allele of the rs2796822 polymorphism and a decreased score in the ACT test, which is associated with worse disease control and the possibility of uncontrolled asthma. The G allele of the rs2796822 polymorphism in the *TGFB2* gene occurs with different frequency in patients with mild and severe asthma. The risk of severe asthma in rs2796822 G allele carriers was 71% higher in comparison to patients without this version of the polymorphism (7).

In a study conducted on a population of Korean asthmatics, 19 SNPs of the *TGFB3* gene were examined using direct re-sequencing (103). A small but statistically significant positive association was found between the methacholine challenge result and the A allele of the +2753G>A polymorphism. No other allele or haplotype examined showed a similar association, indicating that genetic variation at this locus may be partially related to the development of AHR. This study also showed that the strength of the association with nonatopic asthma was significantly greater when the Ht3 (TA) haplotype [consisting of +44T>C (S15F) and +2753G>A] was present rather than when the 2753A allele was present alone (103). The number of patients or families examined is important when drawing conclusions from genetic association studies. Greater statistical power can be achieved by increasing the sample size than by increasing the number of polymorphisms examined. Typically, for candidate gene studies sampling from 500 patients was considered sufficient to provide reproducibility and detect the presence of polymorphisms with a relatively small effect size (104). More recently, candidate gene studies have begun to be replaced by more advanced approaches, such as GWAS using genotyping microarrays or NGS techniques, which require far larger sample sizes. More than 2,000 subjects were recruited for this study (103), which was deemed sufficient for strong reproducibility by the authors.

Additionally, a Pakistani population of 52 asthma patients and 56 healthy controls was genotyped for *TGFβ1* SNPs rs1800469 and rs2241715 (an intron variant) using SNaPshot minisequencing assays. No association between any rs1800469 alleles and asthma occurrence was found. However, under codominant, dominant, over dominant and recessive inheritance models, the T allele of rs2241715 was found to be strongly associated with asthma occurrence, and identified as a risk factor for the Pakistani population (105).A Thai population of 250 patients was examined for the effects of SNPs in the *TGFβ1, ADAM33, VEGFA* and *PLAUR* on asthma occurrence. Interestingly, the *TGFβ1* SNPs examined (rs2241715, rs11466345) were not found to be associated with asthma, while The *ADAM33* rs528557/S2 2151G>C and the *VEGFA* rs833069+450T>G SNPs had a strong positive association with the risk of asthma development (106).

118 children affected by childhood asthma and 120 healthy children in Guangxi, China were genotyped for *TGFβ1* SNPs rs1800469, rs1800470, rs2241712, rs224171 and rs4803455. No significant correlation between any of the SNPs and asthma occurrence was found (107).

The T allele of the rs8179181 polymorphism in an intron of the *TGFB1* gene has been shown to be associated with an increased risk of childhood asthma and atopy in a population of Mexican children and adolescents. It has also been shown to be associated with a more severe course of the disease and elevated levels of *TGFB1* mRNA (108). The A allele of the rs4803455 polymorphism, also in a *TGFB1* intron, was found to be associated with an increased risk of the disease in a study conducted on 380 asthmatics in the Netherlands. In asthma, the presence of this SNP is associated with decreased lung function and a higher degree of airway remodelling (109).

A study conducted on 297 Japanese asthmatic childhood atopic asthma patients revealed an association between a TGF-β2 SNP, and increased risk of childhood atopic asthma. The SNP described in this study was 3′UTR 94862T>A (*p* = 0.00041). The SNP was also found to be associated with a significant increase in eosinophil counts. However, the authors stated that the functional significance of the SNP remains unknown (88).

In summary, genetic variation within the TGF-β genes has been shown to have a definite association with asthma. Variants such as the T allele of the −509C/T and C allele of the +869T/C polymorphisms in *TGFB1* strongly associated with an increased risk of asthma development in several studies. Other variants such as the A allele of the +2753G>A *TGFB3* polymorphism or the G allele of the rs2796822 polymorphism in *TGFB2* have also been shown to influence asthma development, albeit only by single studies carried out on ethnically homogeneous populations. Importantly, the results of studies on −509C/T and +869T/C exhibit strong discrepancies across studies carried out on differing populations, preventing current knowledge on these associations from being applied clinically.

As previously discussed, using the −509C/T polymorphism an example, most studies of asthma-associated alleles show poor reproducibility across genetically diverse populations, which is likely due to variability in the distribution of genetic risk alleles in different ethnicities and differences in the prevalence of gene polymorphisms associated with increased risk of asthma development (110). This lack of reproducibility may reflect genetic heterogeneity underlying the variation in asthma phenotypes. Differences in the allele frequencies of asthma-predisposing genes have been shown between the Chinese population and Central European white, Puerto Rican and Yoruba African populations (111). Additionally, studies carried out on the same polymorphism can lead to disparate results in different age groups, even within the same ethnicity (92).

Table 1 Contains a summary of all the SNPs described here and their association with asthma occurrence.

**Table 1:** Characteristics of single nucleotide polymorphisms (SNPs) of *TGFB* genes studied for association with asthma occurrence.

NCBI SNP ID	Gene	Location	Alleles	Minor allele	MAF	Studied population (ethnicity, mean age ± SD)	Odds ratio, *p* value	Association with asthma
rs1800469	*TGFB1*	promoter	C/T	C	0.497	Polish, 47 ± 16 (60); Hispanic, non-Hispanic white, aged 10–16^a^ (67); Pakistani, aged 4–15^a^ (78); Chinese, children^a^ (80)	(60); 0.98, < 0.05 (67)	Elevated plasma TGFB1 levels, increased risk of bronchial remodelling and asthma development (60, 67)
								No association with asthma (78, 80);
rs1982073	*TGFB1*	exon	C/T	T	0.461	Costa Rican, aged 6–14^a^ (57);	−2.58^b^, 0.009 (57)	Lower risk of asthma exacerbations (57)
rs8179181	*TGFB1*	intron	C/T	T	0.076	Mexican, aged 4–17^a^ (59)	1.48, 0.04 (59)	Increased risk of childhood asthma and atopy, increased disease severity and *TGFB1* mRNA levels (59)
rs4803455	*TGFB1*	intron	C/A	A	0.476	Dutch, no age given (41)	−11.4, 0.03 (41)	Accelerated FEV1 decrease, increased risk of asthma occurrence (41)
rs11083616	*TGFB1*	intron	G/A	A	0.446	Polish, 46 ± 16 (42)	1.85, 0,02 (42)	Increased asthma symptom severity (42)
rs2796821	*TGFB2*	intron	C/T	C	0.291	Polish, 46 ± 16 (42)	1.71, 0.02 (42)	93% increased risk of asthma (heterozygous form), 102% increased risk of asthma (homozygous form) (42)
rs10779329	*TGFB2*	intron	C/T	C	0.421	Polish, 46 ± 16 (42)	1.72, 0,02 (42)	Increased risk of asthma occurrence (42)
rs4903359	*TGFB2*	intron	G/A	G	0.201	Polish, 46 ± 16 (42)	0,74, 0,05 (42)	Increased risk of asthma occurrence (42)
			G/C					
			G/T					
rs8109627	*TGFB1*	intron	C/T	C	0.351	Polish, 48 ± 15 (7)	1,45, 0.01 (7)	Increased ACT scores (7)
rs2796822	*TGFB2*	intron	A/G	A	0.401	Polish, 48 ± 15 (7)	1.97, 0.04 (7)	Decreased ACT scores, 71% increased risk of severe asthma (7)
rs2241715	*TGFB1*	intron	A/C	A	0.341	Pakistani, aged 4–15^a^ (78); Thai, 26 ± 19 (79)	0.24, 0.02 (78)	Increased risk of asthma development (78);
			A/G					
								No association with asthma (79)
rs11466345	*TGFB1*	intron	T/C	C	0.126	Thai, 26 ± 19 (79)		No association with asthma (79)
rs1800470	*TGFB1*	missense	G/A	G	0.427	Chinese, children^a^ (80).		No association with asthma (80)
			G/C					
rs2241712	*TGFB1*	promoter	C/T	C	0.302	Chinese, children^a^ (80).		No association with asthma (80)

MAF, minor allele frequency.

^a^ No mean participant age specified.

^b^ *Z*-score.

## Limitations of current studies, GWAS and future perspectives

Importantly, all of the studies described in this manuscript so far were candidate gene studies or meta-analyses of such studies. This type of studies focuses on specific genes hypothesized to be involved in a particular disease, selected based on established knowledge or biological plausibility. This approach involves examining only a limited number of polymorphisms within the chosen genes of a sample population. It is useful in cases of a known strong association between a gene and a disease, but not for discovering novel associations (112). Indeed, this approach has several limitations. It requires prior knowledge, which limits the rate of novel association discoveries, and can introduce bias. What is more, a small range of hand-picked genes may not reflect the complexity of the pathophysiology and etiology of a disease by not examining the wider genomic context (112).

On the other hand, genome-wide association studies (GWAS) utilise a hypothesis-free approach, and scan the entire genome via modern techniques such as next-generation sequencing (NGS), which has revolutionised the field of genetics. This approach examines millions of SNPs across the whole genome, and usually requires a large study population, which can be difficult to recruit. The major advantage of GWAS is its ability to discover numerous novel associations and create a comprehensive view of the entire genomic complex of a studied disease. Additionally, GWAS can detect variants with small effect sizes or rare variants with larger effects far more efficiently than candidate gene studies (113, 114).

Numerous GWAS have been published on asthma, however most of them do not describe any associations between the disease and TGF-β genes (115–117). Nevertheless, a number of genome-wide studies have found associations of asthma and genes related to TGF-β, or TGF-β genes themselves and other conditions affecting the airways. A GWAS conducted on a UK Biobank, including over 50,000 asthmatic subjects and over 350,000 controls led to the discovery of a causal association between TGFBR1 and asthma development, however no particular gene variants were described (118). Another GWAS described an association between asthma and two SNPs in the GSDMB gene (rs2305480 and rs11078927), which is implicated in TGF-β signalling (119). Several SNPs of SMAD3 (rs16950687, rs1729363, rs744910) the protein product of which is a downstream signalling component of TGF-β and important for regulatory T-cell and TH17 cell pathways, have been associated with asthma in several GWAS (120–122). Importantly, in a GWAS conducted on Mexican subjects with childhood asthma, rs2241715 in TGFB1 has been associated with an increased risk of asthma development. However, the study included only 492 subjects, which is not sufficient for a comprehensive GWAS analysis (123). In a 2011 GWAS conducted on 48,201 European subjects, TGFB2 was found to be a locus strongly associated with pulmonary function (124).

Lastly, two GWAS analyses have found associations between chronic obstructive pulmonary disease (COPD) and loci within TGFB2 (125, 126).

However, despite the numerous candidate gene studies and several GWAS linking TGF-β and related genes to asthma, so far this data has not been applied clinically. In general despite many novel asthma target genes being identified recently, not many of these discoveries have been used in the clinical environment. A major hurdle in applying GWAS findings is the unclear relationship between the novel gene-disease associations and biological mechanisms underlying them (117). Identification of drug targets, such as classical surface receptors and genomics-based drug repositioning, for drugs such as soproxil, olamkicept or inarigivir have been proposed as potential application of GWAS data in asthma (117). However, much GWAS data is still missing for asthma. Many asthma-related genes are missing functional annotations in GWAS, and many cell types relevant to asthma have not had expression quantitative trait locus (eQTL) mapping carried out. Mechanistic links between SNPs identified through GWAS and gene expression in asthma have to be established and validated in cell types involved in asthma pathogenesis (117).

Epigenetic studies are another recent approach used to study relationships between asthma, pharmacological interventions and the human epigenome. These studies have improved our understanding of the molecular basis of treatment response to corticosteroids, bronchodilators and immunotherapy, and how those pharmaceuticals interact with immune and inhibitory pathways, such as IL-2, TNF-α, NF-*κ*B, and C/EBPs (127). Histone modifications are another layer of the epigenome, for which associations with allergic phenotypes, including those related to asthma, were discovered (128).

Importantly, several of these studies have discovered associations with TGF-β signalling pathways. In an epigenetic study of nasal epithelia taken from asthmatic Puerto Rican children, epigenomic responses to albuterol treatment in 22 genome-wide significant CpGs were characterised. TGFB1 was found to be one of the genes, the methylation patterns of which were influenced by albuterol treatment (129). RUNX3, a gene involved in TGF-β signalling, was found to have disparate methylation patterns in peripheral blood leukocytes from children with atopic asthma and non-asthmatic children (130). A genome-wide DNA methylation study examining leukocytes from asthmatic Latino children identified associations between epigenetic regulation of leukocytes and inflammatory signalling pathways. The strongest associations in this study were found for the TGF-β signalling pathway (131). A comprehensive evaluation of these epigenetic associations has the potential to contribute significantly to precision medicine development and new therapeutic target discovery. What is more, epigenetic patterns (including those of the TGF-β pathways) may provide novel tools in the diagnosis of allergic disorders and asthma.

## Conclusions

Asthma exacerbations remain a significant burden for patients, especially young children (132–135). The TGF-β gene polymorphisms discussed in this review show potential as diagnostic factors. In particular, T allele of the −509C/T and C allele of the +869T/C polymorphisms in *TGFB1* were shown to strongly associate with the risk of asthma development. Diagnostic use of these polymorphisms may facilitate earlier and more accurate diagnosis of asthma, allowing appropriate therapy to be initiated and the frequency of exacerbations to be reduced. However, nearly all of the studies published on the associations between TGF-β gene polymorphisms are based on candidate gene studies, and the representation of different ethnic groups should be improved. More studies based on robust and modern methods such as GWAS or NGS techniques, and focused on both genomic and epigenomic associations, need to be carried out on genetically diverse populations to provide stronger evidence and apply TGF-β gene polymorphisms in clinical practice as asthma development risk biomarkers.

## References

[1] Peng D Fu M Wang M Wei Y Wei X. Targeting TGF-β signal transduction for fibrosis and cancer therapy. Mol Cancer. (2022) 21(1):104. 10.1186/s12943-022-01569-x 35461253 PMC9033932

[2] Hata A Chen YG. TGF-β signaling from receptors to smads. Cold Spring Harb Perspect Biol. (2016) 8(9):a022061. 10.1101/cshperspect.a022061 27449815 PMC5008074

[3] Vander Ark A Cao J Li X. TGF-β receptors: in and beyond TGF-β signaling. Cell Signal. (2018) 52:112–20. 10.1016/j.cellsig.2018.09.002 30184463

[4] Hackett TL Warner SM Stefanowicz D Shaheen F Pechkovsky DV Murray LA Induction of epithelial–mesenchymal transition in primary airway epithelial cells from patients with asthma by transforming growth factor-β1. Am J Respir Crit Care Med. (2009) 180(2):122–33. 10.1164/rccm.200811-1730OC 19406982

[5] Xie S Sukkar MB Issa R Oltmanns U Nicholson AG Chung KF. Regulation of TGF-β1-induced connective tissue growth factor expression in airway smooth muscle cells. Am J Physiol Lung Cell Mol Physiol. (2005) 288(1):L68–76. 10.1152/ajplung.00156.2004 15377500

[6] Sidhu SS Yuan S Innes AL Kerr S Woodruff PG Hou L Roles of epithelial cell-derived periostin in TGF-β activation, collagen production, and collagen gel elasticity in asthma. Proc Natl Acad Sci U S A. (2010) 107(32):14170–5. 10.1073/pnas.1009426107 20660732 PMC2922596

[7] Michał P Konrad S Piotr K. TGF-β gene polimorphisms as risk factors for asthma control among clinic patients. J Inflamm. (2021) 18(1):28. 10.1186/s12950-021-00294-4 PMC8499525 34620181

[8] Wnuk D Paw M Ryczek K Bochenek G Sładek K Madeja Z Enhanced asthma-related fibroblast to myofibroblast transition is the result of profibrotic TGF-β/Smad2/3 pathway intensification and antifibrotic TGF-β/Smad1/5/(8)9 pathway impairment. Sci Rep. (2020) 10(1):16492. 10.1038/s41598-020-73473-7 33020537 PMC7536388

[9] Michalik M Wójcik-Pszczoła K Paw M Wnuk D Koczurkiewicz P Sanak M Fibroblast-to-myofibroblast transition in bronchial asthma. Cell Mol Life Sci. (2018) 75(21):3943–61. 10.1007/s00018-018-2899-4 30101406 PMC6182337

[10] Tatler AL John AE Jolly L Habgood A Porte J Brightling C Integrin αvβ5-mediated TGF-β activation by airway smooth muscle cells in asthma. J Immunol. (2011) 187(11):6094–107. 10.4049/jimmunol.1003507 22025551 PMC3242066

[11] Rosendahl A Checchin D Fehniger TE ten Dijke P Heldin CH Sideras P. Activation of the TGF-β/activin-Smad2 pathway during allergic airway inflammation. Am J Respir Cell Mol Biol. (2001) 25(1):60–8. 10.1165/ajrcmb.25.1.4396 11472976

[12] Sagara H Okada T Okumura K Ogawa H Ra C Fukuda T Activation of TGF-β/Smad2 signaling is associated with airway remodeling in asthma. J Allergy Clin Immunol. (2002) 110(2):249–54. 10.1067/mai.2002.126078 12170265

[13] Luo X Ding Q Wang M Li Z Mao K Sun B *In vivo* disruption of TGF-β signaling by Smad7 in airway epithelium alleviates allergic asthma but aggravates lung carcinogenesis in mouse. PLoS One. (2010) 5(4):e10149. 10.1371/journal.pone.0010149 20405019 PMC2854155

[14] Deng Z Fan T Xiao C Tian H Zheng Y Li C TGF-β signaling in health, disease, and therapeutics. Sig Transduct Target Ther. (2024) 9(1):1–40. 10.1038/s41392-023-01668-1 PMC10958066 38514615

[15] Tang J Liu F Cooper ME Chai Z. Renal fibrosis as a hallmark of diabetic kidney disease: potential role of targeting transforming growth factor-beta (TGF-β) and related molecules. Expert Opin Ther Targets. (2022) 26(8):721–38. 10.1080/14728222.2022.2133698 36217308

[16] Wójcik-Pszczoła K Jakieła B Plutecka H Koczurkiewicz P Madeja Z Michalik M Connective tissue growth factor regulates transition of primary bronchial fibroblasts to myofibroblasts in asthmatic subjects. Cytokine. (2018) 102:187–90. 10.1016/j.cyto.2017.09.002 28927757

[17] Stumm CL Halcsik E Landgraf RG Camara NOS Sogayar MC Jancar S. Lung remodeling in a mouse model of asthma involves a balance between TGF-β1 and BMP-7. PLoS One. (2014) 9(4):e95959. 10.1371/journal.pone.0095959 24781156 PMC4004563

[18] GBD 2019 Diseases and Injuries Collaborators. Global burden of 369 diseases and injuries in 204 countries and territories, 1990–2019: a systematic analysis for the global burden of disease study 2019. Lancet. (2020) 396:120–2. 10.1016/S0140-6736(20)30925-9 33069326 PMC7567026

[19] Kuruvilla ME Lee FEH Lee GB. Understanding asthma phenotypes, endotypes and mechanisms of disease. Clin Rev Allerg Immunol. (2019) 56:219–133. 10.1007/s12016-018-8712-1 30206782 PMC6411459

[20] Lötvall J Akdis CA Bacharier LB Bjermer L Casale TB Custovic A Asthma endotypes: a new approach to classification of disease entities within the asthma syndrome. J Allergy Clin Immunol. (2011) 127:355–60. 10.1016/j.jaci.2010.11.037 21281866

[21] Ozdemir C Kucuksezer UC Akdis M Akdis CA. The concepts of asthma endotypes and phenotypes to guide current and novel treatment strategies. Expert Rev Respir Med. (2018) 12:733–43. 10.1080/17476348.2018.1505507 30084271

[22] Lambrecht BN Hammad H. The immunology of asthma. Nat Immunol. (2015) 16(1):45–56. 10.1038/ni.3049 25521684

[23] Pelaia G Vatrella A Busceti MT Gallelli L Calabrese C Terracciano R Cellular mechanisms underlying eosinophilic and neutrophilic airway inflammation in asthma. Mediators Inflamm. (2015) 2015:879783. 10.1155/2015/879783 25878402 PMC4386709

[24] Annunziato F Romagnani C Romagnani S. The 3 major types of innate and adaptive cell-mediated effector immunity. J Allergy Clin Immunol. (2015) 135:626–35. 10.1016/j.jaci.2014.11.001 25528359

[25] Halwani R Al-Muhsen S Al-Jahdali H Hamid Q. Role of transforming growth factor-β in airway remodeling in asthma. Am J Respir Cell Mol Biol. (2011) 44:127–33. 10.1165/rcmb.2010-0027TR 20525803

[26] Chiu CJ Huang MT. Asthma in the precision medicine era: biologics and probiotics. Int J Mol Sci. (2021) 22(9):4528. 10.3390/ijms22094528 33926084 PMC8123613

[27] Reddel HK FitzGerald JM Bateman ED Bacharier LB Becker A Brusselle G GINA 2019: a fundamental change in asthma management: treatment of asthma with short-acting bronchodilators alone is no longer recommended for adults and adolescents. Eur Resp J. (2019) 53:1901046. 10.1183/13993003.01046-2019 31249014

[28] Jia CE Zhang HP Lv Y Liang R Jiang YQ Powell H The asthma control test and asthma control questionnaire for assessing asthma control: systematic review and meta-analysis. J Allergy Clin Immunol. (2013) 131:695–703. 10.1016/j.jaci.2012.08.023 23058645

[29] Anderson GP. Endotyping asthma: new insights into key pathogenic mechanisms in a complex, heterogeneous disease. Lancet. (2008) 372(9643):1107–19. 10.1016/S0140-6736(08)61452-X 18805339

[30] García-Menaya JM Cordobés-Durán C García-Martín E Agúndez JAG. Pharmacogenetic factors affecting asthma treatment response. Potential implications for drug therapy. Front Pharmacol. (2019) 10:520. 10.3389/fphar.2019.00520 31178722 PMC6537658

[31] Slob EMA Vijverberg SJH Palmer CNA Zazuli Z Farzan N Oliveri NMB Pharmacogenetics of inhaled long-acting beta2-agonists in asthma: a systematic review. Pediatr Allergy Immunol. (2018) 29(7):705–14. 10.1111/pai.12956 29992699

[32] Perez-Garcia J Espuela-Ortiz A Lorenzo-Diaz F Pino-Yanes M. Pharmacogenetics of pediatric asthma: current perspectives. Pharmgenomics Pers Med. (2020) 13:89–103. 10.2147/PGPM.S201276 32256100 PMC7090194

[33] Caudle KE Klein TE Hoffman JM Muller DJ Whirl-Carrillo M Gong L Incorporation of pharmacogenomics into routine clinical practice: the clinical pharmacogenetics implementation consortium (CPIC) guideline development process. Curr Drug Metab. (2014) 15:209–17. 10.2174/1389200215666140130124910 24479687 PMC3977533

[34] Redington AE Madden J Frew AJ Djukanovic R Roche WR Holgate ST Transforming growth factor-beta 1 in asthma. Measurement in bronchoalveolar lavage fluid. Am J Respir Crit Care Med. (1997) 156(2 Pt 1):642–7. 10.1164/ajrccm.156.2.9605065 9279252

[35] Batra V Musani AI Hastie AT Khurana S Carpenter KA Zangrilli JG Bronchoalveolar lavage fluid concentrations of transforming growth factor (TGF)-β1, TGF-β2, interleukin (IL)-4 and IL-13 after segmental allergen challenge and their effects on *α*-smooth muscle actin and collagen III synthesis by primary human lung fibroblasts. Clin Exp Allergy. (2004) 34(3):437–44. 10.1111/j.1365-2222.2004.01885.x 15005738

[36] Vignola AM Chanez P Chiappara G Merendino A Pace E Rizzo A Transforming growth factor-beta expression in mucosal biopsies in asthma and chronic bronchitis. Am J Respir Crit Care Med. (1997) 156(2 Pt 1):591–9. 10.1164/ajrccm.156.2.9609066 9279245

[37] Yamaguchi M Niimi A Matsumoto H Ueda T Takemura M Matsuoka H Sputum levels of transforming growth factor-beta1 in asthma: relation to clinical and computed tomography findings. J Investig Allergol Clin Immunol. (2008) 18(3):202–6. 18564632

[38] Naguib M Abd-Elsalam M Abd El-Azeem A Yousif Y. Association between transforming growth factor-β1 and interleukin-4 and childhood asthma in zagazig university hospitals. Zagazig Univ Med J. (2016) 22(5):1–6. 10.21608/zumj.2016.4669

[39] Balzar S Chu HW Silkoff P Cundall M Trudeau JB Strand M Increased TGF-β2 in severe asthma with eosinophilia. J Allergy Clin Immunol. (2005) 115(1):110–7. 10.1016/j.jaci.2004.09.034 15637555

[40] Chu HW Trudeau JB Balzar S Wenzel SE. Peripheral blood and airway tissue expression of transforming growth factor β by neutrophils in asthmatic subjects and normal control subjects. J Allergy Clin Immunol. (2000) 106(6):1115–23. 10.1067/mai.2000.110556 11112895

[41] Eap R Jacques E Semlali A Plante S Chakir J. Cysteinyl leukotrienes regulate TGF-β1 and collagen production by bronchial fibroblasts obtained from asthmatic subjects. Prostaglandins Leukot Essent Fatty Acids. (2012) 86(3):127–33. 10.1016/j.plefa.2011.11.001 22316690

[42] Chakir J Shannon J Molet S Fukakusa M Elias J Laviolette M Airway remodeling-associated mediators in moderate to severe asthma: effect of steroids on TGF-β, IL-11, IL-17, and type I and type III collagen expression. J Allergy Clin Immunol. (2003) 111(6):1293–8. 10.1067/mai.2003.1557 12789232

[43] Firszt R Francisco D Church TD Thomas JM Ingram JL Kraft M. Interleukin-13 induces collagen type-1 expression through matrix metalloproteinase-2 and transforming growth factor-β1 in airway fibroblasts in asthma. Eur Respir J. (2014) 43(2):464–73. 10.1183/09031936.00068712 23682108 PMC6747688

[44] Hassan N Mohamed-Hussein A Mohamed E Mohamed O Mohamed H Tammam M. Serum transforming growth factor –β1 (TGF-β1) in asthmatics: association between disease control, severity and duration. 52 Monitoring Airway Disease (2015), European Respiratory Society, p. OA1465

[45] Khalil F Mohamed NAEG El-Yazed EA. Studying the correlation between transforming growth factor [beta]1 and chitinase-3-like-1 in assessment of bronchial asthma severity. Kasr Al Ainy Medical Journal. (2018) 24(1):7. 10.4103/kamj.kamj_35_17

[46] Lotfy AM Khalil F Zidan H Hadad M Elsayed MA Al-Sayyad MM Assessment of severity of bronchial asthma by studying new markers: transforming growth factor-β1 and chitinase-3-like-1. Egypt J Intern Med. (2016) 28(4):155–61. 10.4103/1110-7782.203295

[47] Ohno I Nitta Y Yamauchi K Hoshi H Honma M Woolley K Transforming growth factor beta 1 (TGF beta 1) gene expression by eosinophils in asthmatic airway inflammation. Am J Respir Cell Mol Biol. (1996) 15(3):404–9. 10.1165/ajrcmb.15.3.8810646 8810646

[48] Ojiaku CA Cao G Zhu W Yoo EJ Shumyatcher M Himes BE TGF-β1 Evokes human airway smooth muscle cell shortening and hyperresponsiveness via Smad3. Am J Respir Cell Mol Biol. (2018) 58(5):575–84. 10.1165/rcmb.2017-0247OC 28984468 PMC5946330

[49] McMillan SJ Xanthou G Lloyd CM. Manipulation of allergen-induced airway remodeling by treatment with anti-TGF-β antibody: effect on the smad signaling pathway1. J Immunol. (2005) 174(9):5774–80. 10.4049/jimmunol.174.9.5774 15843580

[50] Bottoms SE Howell JE Reinhardt AK Evans IC McAnulty RJ. TGF-β isoform specific regulation of airway inflammation and remodelling in a murine model of asthma. PLoS One. (2010) 5(3):e9674. 10.1371/journal.pone.0009674 20300191 PMC2837347

[51] Torrego A Hew M Oates T Sukkar M Chung KF. Expression and activation of TGF-β isoforms in acute allergen-induced remodelling in asthma. Thorax. (2007) 62(4):307–13. 10.1136/thx.2006.063487 17251317 PMC1892798

[52] Wang J Faiz A Ge Q Vermeulen CJ Van der Velden J Snibson KJ Unique mechanisms of connective tissue growth factor regulation in airway smooth muscle in asthma: relationship with airway remodelling. J Cell Mol Med. (2018) 22(5):2826–37. 10.1111/jcmm.13576 29516637 PMC5908101

[53] Altraja S Jaama J Altraja A. Proteome changes of human bronchial epithelial cells in response to pro-inflammatory mediator leukotriene E4 and pro-remodelling factor TGF-β1. J Proteomics. (2010) 73(6):1230–40. 10.1016/j.jprot.2010.02.017 20219718

[54] Tran DQ. TGF-β: the sword, the wand, and the shield of FOXP3+ regulatory T cells. J Mol Cell Biol. (2012) 4:29–37. 10.1093/jmcb/mjr033 22158907

[55] Bettelli E Carrier Y Gao W Korn T Strom TB Oukka M Reciprocal developmental pathways for the generation of pathogenic effector TH17 and regulatory T cells. Nature. (2006) 441(7090):235–8. 10.1038/nature04753 16648838

[56] Mangan PR Harrington LE O’Quinn DB Helms WS Bullard DC Elson CO Transforming growth factor-beta induces development of the T(H)17 lineage. Nature. (2006) 441(7090):231–4. 10.1038/nature04754 16648837

[57] Chen W Ten Dijke P. Immunoregulation by members of the TGFβ superfamily. Nat Rev Immunol. (2016) 16(12):723–40. 10.1038/nri.2016.112 27885276

[58] Saito A Horie M Nagase T. TGF-β signaling in lung health and disease. Int J Mol Sci. (2018) 19(8):2460. 10.3390/ijms19082460 30127261 PMC6121238

[59] Le AV Cho JY Miller M McElwain S Golgotiu K Broide DH. Inhibition of allergen-induced airway remodeling in smad 3-deficient Mice1. J Immunol. (2007) 178(11):7310–6. 10.4049/jimmunol.178.11.7310 17513781

[60] Gregory LG Mathie SA Walker SA Pegorier S Jones CP Lloyd CM. Overexpression of Smad2 drives house dust Mite–mediated airway remodeling and airway hyperresponsiveness via activin and IL-25. Am J Respir Crit Care Med. (2010) 182(2):143–54. 10.1164/rccm.200905-0725OC 20339149 PMC2913231

[61] Flood-Page P Menzies-Gow A Phipps S Ying S Wangoo A Ludwig MS Anti-IL-5 treatment reduces deposition of ECM proteins in the bronchial subepithelial basement membrane of mild atopic asthmatics. J Clin Invest. (2003) 112(7):1029–36. 10.1172/JCI17974 14523040 PMC198522

[62] Mosmann TR Cherwinski H Bond MW Giedlin MA Coffman RL. Two types of murine helper T cell clone. I. Definition according to profiles of lymphokine activities and secreted proteins. J Immunol Baltim Md 1950. (1986) 136(7):2348–57. 2419430

[63] Kim SY Kim TB Moon K Kim TJ Shin D Cho YS Regulation of pro-inflammatory responses by lipoxygenases via intracellular reactive oxygen species *in vitro* and *in vivo*. Exp Mol Med. (2008) 40(4):461–76. 10.3858/emm.2008.40.4.461 18779659 PMC2679273

[64] Wills-Karp M Luyimbazi J Xu X Schofield B Neben TY Karp CL Interleukin-13: central mediator of allergic asthma. Science. (1998) 282(5397):2258–61. 10.1126/science.282.5397.2258 9856949

[65] Lee CG Homer RJ Zhu Z Lanone S Wang X Koteliansky V Interleukin-13 induces tissue fibrosis by selectively stimulating and activating transforming growth factor beta(1). J Exp Med. (2001) 194(6):809–21. 10.1084/jem.194.6.809 11560996 PMC2195954

[66] Panek M Stawiski K Kaszkowiak M Kuna P. Cytokine TGFβ gene polymorphism in asthma: TGF-related SNP analysis enhances the prediction of disease diagnosis (A case-control study with multivariable data-mining model development). Front Immunol. (2022) 13:746360. 10.3389/fimmu.2022.746360 35774789 PMC9238410

[67] EMBL's European Bioinformatics Institute (EMBL-EBI). Gene: TGFB1 (ENSG00000105329)—Summary—Homo_sapiens—Ensembl genome browser 113. Available online at: https://www.ensembl.org/Homo_sapiens/Gene/Summary?g=ENSG00000105329;r=19:41301587-41353922 (Accessed January 11, 2025).

[68] EMBL-EBI. Gene: TGFBR3 (ENSG00000069702)—Summary—Homo_sapiens—Ensembl genome browser 113. Available online at: https://www.ensembl.org/Homo_sapiens/Gene/Summary?db=core;g=ENSG00000069702;r=1:91680343-91906335 (Accessed January 11, 2025).

[69] Aceves SS Newbury RO Chen D Mueller J Dohil R Hoffman H Resolution of remodeling in eosinophilic esophagitis correlates with epithelial response to topical corticosteroids. Allergy. (2010) 65(1):109–16. 10.1111/j.1398-9995.2009.02142.x 19796194 PMC2807896

[70] Nemeth K Keane-Myers A Brown JM Metcalfe DD Gorham JD Bundoc VG Bone marrow stromal cells use TGF-β to suppress allergic responses in a mouse model of ragweed-induced asthma. Proc Natl Acad Sci. (2010) 107(12):5652–7. 10.1073/pnas.0910720107 20231466 PMC2851758

[71] El-Shal AS Shalaby SM Abdel-Nour HM Sarhan WM Hamed Gehad M Mohamed Yousif Y. Impact of cytokines genes polymorphisms and their serum levels on childhood asthma in Egyptian population. Cytokine. (2022) 157:155933. 10.1016/j.cyto.2022.155933 35728502

[72] Verbsky JW Chatila TA. Immune dysregulation, polyendocrinopathy, enteropathy, X-linked (IPEX) and IPEX-related disorders: an evolving web of heritable autoimmune diseases. Curr Opin Pediatr. (2013) 25(6):708–14. 10.1097/MOP.0000000000000029 24240290 PMC4047515

[73] Zhang Y Collier F Naselli G Saffery R Tang MLK Allen KJ Cord blood monocyte-derived inflammatory cytokines suppress IL-2 and induce nonclassic ‘T(H)2-type’ immunity associated with development of food allergy. Sci Transl Med. (2016) 8(321):321ra8. 10.1126/scitranslmed.aad4322 26764159

[74] Chen W Jin W Hardegen N Lei K Li L Marinos N Conversion of peripheral CD4+ CD25− naive T cells to CD4+ CD25+ regulatory T cells by TGF-β induction of transcription factor Foxp3. J Exp Med. (2003) 198(12):1875–86. 10.1084/jem.20030152 14676299 PMC2194145

[75] Mendoza RP Anderson CC Fudge DH Roede JR Brown JM. Metabolic consequences of IgE- and non-IgE–mediated mast cell degranulation. J Immunol. (2021) 207(11):2637–48. 10.4049/jimmunol.2001278 34732470 PMC8612977

[76] Hobbs K Negri J Klinnert M Rosenwasser LJ Borish L. Interleukin-10 and transforming growth factor- β promoter polymorphisms in allergies and asthma. Am J Respir Crit Care Med. (1998) 158(6):1958–62. 10.1164/ajrccm.158.6.9804011 9847292

[77] Grainger DJ Heathcote K Chiano M Snieder H Kemp PR Metcalfe JC Genetic control of the circulating concentration of transforming growth factor type beta1. Hum Mol Genet. (1999) 8(1):93–7. 10.1093/hmg/8.1.93 9887336

[78] Nagpal K Sharma S B-Rao C Nahid S Niphadkar PV Sharma SK TGFβ1 haplotypes and asthma in Indian populations. J Allergy Clin Immunol. (2005) 115(3):527–33. 10.1016/j.jaci.2004.11.048 15753900

[79] Chiang CH Chuang CH Liu SL Shen HD. Genetic polymorphism of transforming growth factor β1 and tumor necrosis factor *α* is associated with asthma and modulates the severity of asthma. Respir Care. (2013) 58(8):1343–50. 10.4187/respcare.02187 23466425

[80] Yucesoy B Kashon ML Johnson VJ Lummus ZL Fluharty K Gautrin D Genetic variants in TNFα, TGFB1, PTGS1 and PTGS2 genes are associated with diisocyanate-induced asthma. J Immunotoxicol. (2016) 13(1):119–26. 10.3109/1547691X.2015.1017061 25721048 PMC4814713

[81] Li H Li Y Zhang M Xu G Feng X Xi J Associations of genetic variants in ADAM33 and TGF-β1 genes with childhood asthma risk. Biomed Rep. (2014) 2(4):533–8. 10.3892/br.2014.280 24944803 PMC4051479

[82] Ueda T Niimi A Matsumoto H Takemura M Yamaguchi M Matsuoka H TGFB1 promoter polymorphism C-509T and pathophysiology of asthma. J Allergy Clin Immunol. (2008) 121(3):659–64. 10.1016/j.jaci.2007.10.005 18036644

[83] Yang XX Li FX Wu YS Wu D Tan JY Li M. Association of TGF-beta1, IL-4 and IL-13 gene polymerphisms with asthma in a Chinese population. Asian Pac J Allergy Immunol. (2011) 29(3):273–7. 22053598

[84] Lv J Liu Q Hua L Dong X Bao Y. Association of five single nucleotide polymorphism loci with asthma in children of Chinese han nationality. J Asthma. (2009) 46(6):582–5. 10.1080/02770900902915847 19657898

[85] Weissler KA Frischmeyer-Guerrerio PA. Genetic evidence for the role of transforming growth factor-β in atopic phenotypes. Curr Opin Immunol. (2019) 60:54–62. 10.1016/j.coi.2019.05.002 31163387 PMC6800617

[86] Silverman ES Palmer LJ Subramaniam V Hallock A Mathew S Vallone J Transforming growth factor-beta1 promoter polymorphism C-509T is associated with asthma. Am J Respir Crit Care Med. (2004) 169(2):214–9. 10.1164/rccm.200307-973OC 14597484

[87] Wiśniewski A Obojski A Pawlik A Jasek M Luszczek W Majorczyk E Polymorphism of the TGFB1 gene is not associated with bronchial allergic asthma in a Polish population. Hum Immunol. (2009) 70(2):134–8. 10.1016/j.humimm.2008.12.002 19136038

[88] Hatsushika K Hirota T Harada M Sakashita M Kanzaki M Takano S Transforming growth factor-β2 polymorphisms are associated with childhood atopic asthma. Clin Exp Allergy. (2007) 37(8):1165–74. 10.1111/j.1365-2222.2007.02768.x 17651146

[89] Panek M Jonakowski M Zioło J Wieteska Ł Małachowska B Pietras T A novel approach to understanding the role of polymorphic forms of the NR3C1 and TGF-β1 genes in the modulation of the expression of IL-5 and IL-15 mRNA in asthmatic inflammation. Mol Med Rep. (2016) 13(6):4879–87. 10.3892/mmr.2016.5104 27081784

[90] Salam MT Gauderman WJ McConnell R Lin PC Gilliland FD. Transforming growth factor-β1 C-509T polymorphism, oxidant stress, and early-onset childhood asthma. Am J Respir Crit Care Med. (2007) 176(12):1192–9. 10.1164/rccm.200704-561OC 17673695 PMC2176104

[91] de Faria ICJ de Faria EJ Toro AADC Ribeiro JD Bertuzzo CS. Association of TGF-beta1, CD14, IL-4, IL-4R and ADAM33 gene polymorphisms with asthma severity in children and adolescents. J Pediatr (Rio J). (2008) 84(3):203–10. 10.2223/JPED.1783 18425216

[92] Liu Z Li J Wang K Tan Q Tan W Guo G. Association between TGF-β1 polymorphisms and asthma susceptibility among the Chinese: a meta-analysis. Genet Test Mol Biomark. (2018) 22(7):433–42. 10.1089/gtmb.2017.0238 29958018

[93] Shah R Hurley CK Posch PE. A molecular mechanism for the differential regulation of TGF-beta1 expression due to the common SNP −509C-T (c. −1347C>T). Hum Genet. (2006) 120(4):461–9. 10.1007/s00439-006-0194-1 16896927

[94] Pulleyn LJ Newton R Adcock IM Barnes PJ. TGFbeta1 allele association with asthma severity. Hum Genet. (2001) 109(6):623–7. 10.1007/s00439-001-0617-y 11810274

[95] Yao YS Chang WW He LP Jin YL Li CP. An updated meta-analysis of transforming growth factor-β1 gene: three well-characterized polymorphisms with asthma. Hum Immunol. (2016) 77(12):1291–9. 10.1016/j.humimm.2016.09.011 27717847

[96] Sharma S Raby BA Hunninghake GM Soto-Quirós M Avila L Murphy AJ Variants in TGFB1, dust mite exposure, and disease severity in children with asthma. Am J Respir Crit Care Med. (2009) 179(5):356–62. 10.1164/rccm.200808-1268OC 19096005 PMC2648908

[97] di Palmo E Cantarelli E Catelli A Ricci G Gallucci M Miniaci A The predictive role of biomarkers and genetics in childhood asthma exacerbations. Int J Mol Sci. (2021) 22(9):4651. 10.3390/ijms22094651 33925009 PMC8124320

[98] Buckova D Izakovicová Hollá L Benes P Znojil V Vácha J. TGF-beta1 gene polymorphisms. Allergy. (2001) 56(12):1236–7. 10.1034/j.1398-9995.2001.00373.x 11736765

[99] Cox A Dunning AM Garcia-Closas M Balasubramanian S Reed MWR Pooley KA A common coding variant in CASP8 is associated with breast cancer risk. Nat Genet. (2007) 39(3):352–8. 10.1038/ng1981 17293864

[100] Mak JCW Leung HCM Ho SP Law BKW Ho ASS Lam WK Analysis of TGF-beta(1) gene polymorphisms in Hong Kong Chinese patients with asthma. J Allergy Clin Immunol. (2006) 117(1):92–6. 10.1016/j.jaci.2005.08.049 16387590

[101] Dunning AM Ellis PD McBride S Kirschenlohr HL Healey CS Kemp PR A transforming growth factorbeta1 signal peptide variant increases secretion *in vitro* and is associated with increased incidence of invasive breast cancer. Cancer Res. (2003) 63(10):2610–5. 12750287

[102] Yokota M Ichihara S Lin TL Nakashima N Yamada Y. Association of a T29→C polymorphism of the transforming growth factor-β1 gene with genetic susceptibility to myocardial infarction in Japanese. Circulation. (2000) 101(24):2783–7. 10.1161/01.CIR.101.24.2783 10859282

[103] Kim HK Jang TW Jung MH Park HW Lee JE Shin ES Association between genetic variations of the transforming growth factor β receptor type III and asthma in a Korean population. Exp Mol Med. (2010) 42(6):420–7. 10.3858/emm.2010.42.6.043 20386084 PMC2892595

[104] Long AD Langley CH. The power of association studies to detect the contribution of candidate genetic loci to variation in complex traits. Genome Res. (1999) 9(8):720–31. 10.1101/gr.9.8.720 10447507 PMC310800

[105] Akram M Sabar MF Bano I Ghani MU Shahid M. Single nucleotide polymorphisms of transforming growth factor-Β1 gene as potential asthma susceptible variants in Punjabi population of Pakistan. J Ayub Med Coll Abbottabad. (2022) 34(Suppl 1):S944–8. 10.55519/JAMC-04-S4-10495 36550650

[106] Thongngarm T Jameekornrak A Chaiyaratana N Thongnoppakhun W Sangasapaviliya A Jirapongsananuruk O Effect of gene polymorphisms in ADAM33, TGFβ1, VEGFA, and PLAUR on asthma in Thai population. Asian Pac J Allergy Immunol. (2022) 40(1):39–46. 10.12932/AP-010419-0532 31586488

[107] Cao F Lin N Lin J Yang G Wang X. Correlation between TGFβ1 gene polymorphism and asthma in baise, Guangxi children. J Biosci Med. (2024) 12(5):300–11. 10.4236/jbm.2024.125023

[108] Li H Romieu I Wu H Sienra-Monge JJ Ramírez-Aguilar M del Río-Navarro BE Genetic polymorphisms in transforming growth factor beta-1 (TGFB1) and childhood asthma and atopy. Hum Genet. (2007) 121(5):529–38. 10.1007/s00439-007-0337-z 17333284 PMC1865573

[109] Ierodiakonou D Postma DS Koppelman GH Gerritsen J ten Hacken NH Timens W TGF-β1 polymorphisms and asthma severity, airway inflammation, and remodeling. J Allergy Clin Immunol. (2013) 131:582–5. 10.1016/j.jaci.2012.08.013 23111237

[110] Chen Y Wong GW Li J. Environmental exposure and genetic predisposition as risk factors for asthma in China. Allergy Asthma Immunol Res. (2016) 8(2):92–100. 10.4168/aair.2016.8.2.92 26739401 PMC4713885

[111] Leung TF Ko FWS Sy HY Tsui SKW Wong GWK. Differences in asthma genetics between Chinese and other populations. J Allergy Clin Immunol. (2014) 133(1):42–8. 10.1016/j.jaci.2013.09.018 24188974

[112] Patnala R Clements J Batra J. Candidate gene association studies: a comprehensive guide to useful in silico tools. BMC Genet. (2013) 14:39. 10.1186/1471-2156-14-39 23656885 PMC3655892

[113] Amos W Driscoll E Hoffman JI. Candidate genes versus genome-wide associations: which are better for detecting genetic susceptibility to infectious disease? Proc Biol Sci. (2011) 278(1709):1183–8. 10.1098/rspb.2010.1920 20926441 PMC3049081

[114] Tam V Patel N Turcotte M Bossé Y Paré G Meyre D. Benefits and limitations of genome-wide association studies. Nat Rev Genet. (2019) 20(8):467–84. 10.1038/s41576-019-0127-1 31068683

[115] Chang D Hunkapiller J Bhangale T Reeder J Mukhyala K Tom J A whole genome sequencing study of moderate to severe asthma identifies a lung function locus associated with asthma risk. Sci Rep. (2022) 12(1):5574. 10.1038/s41598-022-09447-8 35368043 PMC8976834

[116] Han Y Jia Q Jahani PS Hurrell BP Pan C Huang P Genome-wide analysis highlights contribution of immune system pathways to the genetic architecture of asthma. Nat Commun. (2020) 11(1):1776. 10.1038/s41467-020-15649-3 32296059 PMC7160128

[117] El-Husseini ZW Gosens R Dekker F Koppelman GH. The genetics of asthma and the promise of genomics-guided drug target discovery. Lancet Respir Med. (2020) 8(10):1045–56. 10.1016/S2213-2600(20)30363-5 32910899

[118] Valette K Li Z Bon-Baret V Chignon A Bérubé JC Eslami A Prioritization of candidate causal genes for asthma in susceptibility loci derived from UK biobank. Commun Biol. (2021) 4(1):1–15. 10.1038/s42003-021-02227-6 34103634 PMC8187656

[119] Tulah AS Holloway JW Sayers I. Defining the contribution of SNPs identified in asthma GWAS to clinical variables in asthmatic children. BMC Med Genet. (2013) 14(1):100. 10.1186/1471-2350-14-100 24066901 PMC3849932

[120] Li X Ampleford EJ Howard TD Moore WC Torgerson DG Li H Genome-wide association studies of asthma indicate opposite immunopathogenesis direction from autoimmune diseases. J Allergy Clin Immunol. (2012) 130(4):861–8.e7. 10.1016/j.jaci.2012.04.041 22694930 PMC3579216

[121] Tang MF Sy HY Kong AP Ko FW Wang SS Liu TC Genetic effects of multiple asthma loci identified by genomewide association studies on asthma and spirometric indices. Pediatr Allergy Immunol. (2016) 27(2):185–94. 10.1111/pai.12505 26534891

[122] Moffatt MF Gut IG Demenais F Strachan DP Bouzigon E Heath S A large-scale, consortium-based genomewide association study of asthma. N Engl J Med. (2010) 363(13):1211–21. 10.1056/NEJMoa0906312 20860503 PMC4260321

[123] Wu H Romieu I Shi M Hancock DB Li H Sienra-Monge JJ Evaluation of candidate genes in a genome-wide association study of childhood asthma in Mexicans. J Allergy Clin Immunol. (2010) 125(2):321–7.e13. 10.1016/j.jaci.2009.09.007 19910030 PMC2823974

[124] Soler Artigas M Loth DW Wain LV Gharib SA Obeidat M Tang W Genome-wide association and large-scale follow up identifies 16 new loci influencing lung function. Nat Genet. (2011) 43(11):1082–90. 10.1038/ng.941 21946350 PMC3267376

[125] Busch R Hobbs BD Zhou J Castaldi PJ McGeachie MJ Hardin ME Genetic association and risk scores in a chronic obstructive pulmonary disease meta-analysis of 16,707 subjects. Am J Respir Cell Mol Biol. (2017) 57(1):35–46. 10.1165/rcmb.2016-0331OC 28170284 PMC5516277

[126] Wain LV Shrine N Artigas MS Erzurumluoglu AM Noyvert B Bossini-Castillo L Genome-wide association analyses for lung function and chronic obstructive pulmonary disease identify new loci and potential druggable targets. Nat Genet. (2017) 49(3):416–25. 10.1038/ng.3787 28166213 PMC5326681

[127] Perez-Garcia J Cardenas A Lorenzo-Diaz F Pino-Yanes M. Precision medicine for asthma treatment: unlocking the potential of the epigenome and microbiome. J Allergy Clin Immunol. (2024). 10.1016/j.jaci.2024.06.010 38906272 PMC12002393

[128] Alaskhar Alhamwe B Khalaila R Wolf J von Bülow V Harb H Alhamdan F Histone modifications and their role in epigenetics of atopy and allergic diseases. Allergy Asthma Clin Immunol. (2018) 14:39. 10.1186/s13223-018-0259-4 29796022 PMC5966915

[129] Perez-Garcia J Pino-Yanes M Plender EG Everman JL Eng C Jackson ND Epigenomic response to albuterol treatment in asthma-relevant airway epithelial cells. Clin Epigenetics. (2023) 15:156. 10.1186/s13148-023-01571-0 37784136 PMC10546710

[130] Yang IV Pedersen BS Liu A O’Connor GT Teach SJ Kattan M DNA methylation and childhood asthma in the inner city. J Allergy Clin Immunol. (2015) 136(1):69–80. 10.1016/j.jaci.2015.01.025. 25769910 PMC4494877

[131] Jiang Y Forno E Han YY Xu Z Hu D Boutaoui N A genome-wide study of DNA methylation in white blood cells and asthma in Latino children and youth. Epigenetics. (2021) 16(5):577–85. 10.1080/15592294.2020.1809872 32799603 PMC8078676

[132] Pardue Jones B Fleming GM Otillio JK Asokan I Arnold DH. Pediatric acute asthma exacerbations: evaluation and management from emergency department to intensive care unit. J Asthma. (2016) 53(6):607–17. 10.3109/02770903.2015.1067323 27116362

[133] Herrera-Luis E Hernandez-Pacheco N Vijverberg SJ Flores C Pino-Yanes M. Role of genomics in asthma exacerbations. Curr Opin Pulm Med. (2019) 25(1):101. 10.1097/MCP.0000000000000533 30334825

[134] FitzGerald JM Barnes PJ Chipps BE Jenkins CR O’Byrne PM Pavord ID The burden of exacerbations in mild asthma: a systematic review. ERJ Open Res. (2020) 6(3):00359–2019. 10.1183/23120541.00359-2019 32802826 PMC7418821

[135] Chapman KR. Impact of ‘mild’ asthma on health outcomes: findings of a systematic search of the literature. Respir Med. (2005) 99(11):1350–62. 10.1016/j.rmed.2005.03.020 16210094

